# Integration of multi-omics and machine learning strategies identifies immune related candidate biomarkers in inflammation-associated hypertrophic cardiomyopathy

**DOI:** 10.3389/fimmu.2025.1645382

**Published:** 2025-09-26

**Authors:** Qingzhu Liang, Jin Wang, Qingxiao Nong, Shouwen Tao, Dalang Fang

**Affiliations:** ^1^ Department of Health, Baise People’s Hospital, Baise, Guangxi, China; ^2^ Department of Gland Surgery, Affiliated Hospital of Youjiang Medical University for Nationalities, Key Laboratory of Tumor Molecular Pathology of Baise, Baise, Guangxi, China

**Keywords:** hypertrophic cardiomyopathy, multi-approach, machine learning, biomarkers, immune infiltration

## Abstract

**Background:**

Hypertrophic cardiomyopathy (HCM) is a common inherited heart disease frequently leading to heart failure. Although sarcomeric gene mutations are known, they only account for a subset of cases. The role of immune dysregulation in HCM progression has gained increasing attention, necessitating the exploration of immune-related biomarkers and therapeutic targets. This study integrates Mendelian randomization (MR), transcriptomics, machine learning, and experimental validation to investigate the immune mechanisms underlying HCM.

**Methods:**

We analyzed three transcriptomic datasets from the GEO database (210 healthy controls, 152 HCM patients) and identified differentially expressed genes (DEGs) using the R package limma. MR analysis was performed on 19,942 expression quantitative trait loci (eQTLs) and HCM cases using the TwoSampleMR package. Machine learning (10 algorithms) was employed to construct diagnostic models, and SHAP analysis was applied to assess key gene contributions. Functional enrichment was performed with clusterProfiler, diagnostic performance was evaluated via ROC curves, and immune cell infiltration was analyzed using CIBERSORT. A competing endogenous RNA (ceRNA) network was constructed, and drug targets were predicted via the DGIdb database. Key gene expression was validated by qPCR.

**Results:**

We identified 472 DEGs and 205 HCM-associated loci, narrowing down to seven key genes: RNF165, SNCA, SRGN, MARCO, STEAP4, SIGLEC9, and TKT. These genes were enriched in immune-related pathways (e.g., cytokine activity, leukocyte migration, JAK-STAT signaling). The Random Forest model exhibited the highest diagnostic performance (AUC: 0.939), with SHAP analysis revealing MARCO as the top contributor. Gene expression was associated with immune cell infiltration: HCM samples showed increased CD4+ T cells and M0 macrophages but decreased M2 macrophages and neutrophils. The ceRNA network comprised 5 mRNAs, 40 miRNAs, and 152 lncRNAs. SRGN and SNCA were identified as potential targets for heparin and 33 other drugs, respectively. qRT-PCR performed on a small number of myocardial samples supported expression trends of the identified genes, in line with transcriptomic analysis.

**Conclusion:**

This study reveals immune-related mechanistic biomarkers and potential therapeutic targets in HCM, highlighting the role of immune dysregulation in disease progression. Machine learning and SHAP analysis improved diagnostic model interpretability, providing a basis for future development of non-invasive diagnostic tools.

## 1 Introduction

Hypertrophic cardiomyopathy (HCM), a heterogeneous monogenic cardiac disorder studied for over five decades, is recognized as a leading cause of arrhythmic sudden death, heart failure, and atrial fibrillation (with thromboembolic stroke) (1). Epidemiologic studies across diverse populations—including the United States, Europe, Japan, China, and East Africa—have established HCM as the most prevalent inherited cardiomyopathy, affecting at least 1 in 500 individuals (0.2%) in the general population (2–4). Extrapolated estimates suggest approximately 600,000 affected individuals in the United States and 120,000 in the United Kingdom, though these figures likely underestimate disease burden due to undiagnosed familial cases. The molecular pathogenesis of HCM is driven by over 1,400 mutations in 11 or more genes encoding sarcomeric proteins. Despite its status as the most common cause of sudden cardiac death in young individuals (including trained athletes) and its association with functional disability from heart failure or stroke, most affected individuals remain undiagnosed due to asymptomatic presentations, with many experiencing preserved life expectancy and minimal symptoms. Clinical diagnosis relies on imaging evidence of unexplained left ventricular hypertrophy (LVH) by echocardiography or cardiovascular magnetic resonance (CMR). Notably, an emerging subgroup harbors pathogenic mutations without LVH, whose natural history remains undefined. Over decades of research, HCM has evolved from a rare, untreatable entity to a common genetic condition with management strategies capable of restoring quality of life and extending survival (5).

Classically defined as a monogenic disorder with autosomal dominant inheritance, HCM exhibits marked temporal and phenotypic heterogeneity. However, prevailing research has predominantly focused on sarcomeric gene mutations and their impact on cardiomyocyte contractility, despite these variants being identified in only a minority of patients. The Hypertrophic Cardiomyopathy Registry (HCMR) delineated two distinct cohorts: genotype-positive patients with confirmed sarcomere mutations and genotype-negative individuals (6). Significant differences in morphological features, fibrosis burden, and dynamic obstruction on CMR underscore the necessity to integrate clinical and imaging phenotypes with circulating biomarkers that reflect disease activity. Such biomarkers hold promise for improving diagnosis, guiding therapy, and predicting outcomes. Disappointingly, genome-wide sequencing studies have largely failed to identify novel pathogenic mutations beyond sarcomeric genes, instead yielding variants of uncertain significance (VUS), highlighting the limitations of a purely monogenic framework.

In the post-genomic era, critical challenges persist in optimizing patient selection for genetic testing and enhancing diagnostic yield. The Mayo Clinic HCM Genotype Predictor Score, widely used in clinical practice, estimates pretest probability of a pathogenic variant by incorporating echocardiographic LV characteristics, age at diagnosis, and family history. Yet, even with this algorithm, the overall positivity rate of genetic testing remains modest at 34% (7). This gap underscores the likelihood of polygenic contributions to HCM pathogenesis, necessitating collaborative efforts among clinicians, geneticists, and molecular biologists to unravel multifactorial mechanisms. A seminal review by Chou et al. (8) advances this paradigm by proposing an integrative model that extends beyond sarcomere-centric pathophysiology. Their work elucidates how calcium dysregulation, impaired autophagy, and metabolic perturbations intrinsically drive cardiomyocyte hypertrophy. Complementary to genomic and transcriptomic approaches, proteomic and phosphoproteomic analyses (9, 10) offer unique insights into post-translational modifications that may underlie phenotypic diversity. The application of machine learning (ML) and artificial intelligence (AI) in genomic disease diagnosis offers new possibilities for addressing complex challenges in HCM diagnosis. These technologies can integrate large-scale genomic, transcriptomic, and phenotypic data to identify polygenic interactions and nonlinear patterns that are difficult to detect using conventional methods. By developing predictive models, machine learning algorithms can significantly improve the detection efficiency of pathogenic variants, compensating for limitations in diagnostic yield seen in traditional scoring systems.

Although hypertrophic cardiomyopathy (HCM) has traditionally been classified as a non-inflammatory cardiomyopathy, growing transcriptomic and histopathologic evidence indicates that immune cell infiltration, cytokine imbalance, and maladaptive inflammatory responses may contribute to disease progression, myocardial fibrosis, and arrhythmogenic remodeling (11, 12). Specifically, pro-inflammatory signaling pathways such as NF-kB and interferon responses have been implicated in cardiomyocyte stress, while altered expression of immune-related genes involved in macrophage activation, T-cell signaling, and antigen presentation has been observed in hypertrophied myocardium (13, 14). Accumulation of macrophages and interactions between fibroblasts and immune cells may drive interstitial fibrosis and increased myocardial stiffness, both characteristic features of advancing HCM (15). In addition, studies utilizing single-cell RNA sequencing and immune deconvolution algorithms such as CIBERSORT have revealed shifts in immune cell composition, including increased infiltration of M2 macrophages and regulatory T cells, suggesting a potential immunoregulatory role in cardiac remodeling (16). Although HCM is classically described as a monogenic disorder, pathogenic variants are identifiable in only 40-60% of patients (17), highlighting the presence of additional molecular contributors. Emerging evidence suggests that chronic low-grade inflammation and myocardial fibrosis coexist in HCM and may contribute to disease progression independently of sarcomere gene mutations (18, 19). Recent single-cell and spatial transcriptomic studies have revealed complex immune cell heterogeneity and inflammatory signaling within cardiac tissues (20, 21), underscoring the need to elucidate immune mechanisms in cardiomyopathies. These findings support the rationale for an integrated transcriptomic and immunogenomic analysis to identify immune-related biomarkers and potential therapeutic targets in HCM.

Building upon these findings, our study employs GWAS analysis integrated with transcriptomic profiling and experimental validation to identify high-risk molecular markers for HCM. To further enhance diagnostic accuracy and deepen understanding of molecular mechanisms, we incorporated machine learning methods and SHAP analysis to evaluate the diagnostic potential of key genes and their role in HCM immune regulation. To date, few studies have systematically integrated transcriptomics, causal inference, and machine learning to explore immune mechanisms in HCM (22). Our work provides a novel framework by combining Mendelian randomization, multi-dataset transcriptome integration, SHAP-interpretable machine learning models, and ceRNA/drug-target network analysis. This strategy not only identifies immune-related genes associated with HCM but also reveals potential regulatory and therapeutic pathways, offering new insights into the immunogenomic landscape of this disease. Recent evidence suggests that immune cell infiltration, inflammatory signaling, and interactions between immune cells and cardiac fibroblasts may promote myocardial fibrosis and structural remodeling (23). By incorporating immunogenomic features into our analysis, we aim to improve interpretation of genotype-phenotype heterogeneity and reveal molecular mechanisms beyond the traditional sarcomere model. Ultimately, this approach is expected to improve risk stratification for HCM patients and guide precision treatment strategies.

## 2 Materials and methods

### 2.1 Raw data

We obtained three transcriptome datasets from the GEO database, GSE141910 (left ventricular samples from 166 healthy controls and 28 HCM), GSE160997 (left ventricular samples from 5 healthy controls and 18 HCM), and GSE36961 (left ventricular samples from 39 healthy controls and 106 HCM). Only samples explicitly labeled as hypertrophic cardiomyopathy (HCM) were included in our study. Samples from other cardiomyopathy subtypes, such as dilated or restrictive cardiomyopathy, were excluded during the data curation and preprocessing stage to ensure cohort purity (see **Supplementary Table S1**). We used the “sva” package (https://bioconductor.org/packages/release/bioc/html/sva.html) to merge and normalize the three datasets, then performed differentially expressed gene analysis between HCM and control samples using the limma package (https://bioconductor.org/packages/release/bioc/html/limma.html) of R software (Version 4.3.1). The screening thresholds for differentially expressed genes were defined as an absolute value of log2 fold change |logFC| > 0.5 and a false discovery rate (FDR) < 0.05.

To ensure cross-platform comparability, raw expression matrices were preprocessed using platform-specific normalization methods. For RNA-seq datasets, raw counts were transformed into TPM values and log2-transformed. For the microarray dataset (GSE36961), we applied robust multi-array average (RMA) normalization. The normalized matrices were then merged, and the “ComBat” function from the sva package was used to correct for batch effects across datasets, which is widely used and validated for cross-platform integration. Principal component analysis (PCA) was performed before and after correction to visually confirm the reduction of batch-related variance.

### 2.2 Screening of expression quantitative trait locus exposure data and HCM outcome data

The function “extract_instruments” in the R package “TwoSampleMR” (https://github.com/MRCIEU/TwoSampleMR) were utilized to summarize SNPs data of 19942 eQTLs from the GWAS database. The filtering criteria for SNPs were as follows: p < 5e-08, clump_r2 = 0.001, clump_Kb =10000. The HCM outcome data for this study consisted of a total of 24,199,797 SNPs obtained from 507 HCM samples and 489,220 control samples from European with the GWAS ID: ebi-a-GCST90018861.

### 2.3 Two-sample GWAS analysis between exposure data and outcome data

The MR analysis followed three basic assumptions, Specifically, to be used as an instrumental variable for a risk factor, a genetic variant or variants must satisfy (1): be reliably associated with the risk factor under study (association hypothesis) (2); no association with any known or unknown confounders (independence assumption) (3); affecting the outcome only through the risk factor and not through any other direct causal pathway (excluding limiting assumptions). We applied five complementary two-sample MR Methods: inverse variance weighting (IVW), MR-Egger, weighted median (WM), weighted modal, and simple modal methods using the R package TwoSampleMR for exposure data eQTL and outcome data HCM. We employed the IVW method with a significance threshold of p<0.05 and Mendelian analysis pleiotropy with p values >0.05 as significance thresholds to screen for criteria in order to identify candidate eQTLs.

### 2.4 Screening key biomarkers

The MR Analysis results were subjected to IVW<0.05 screening to identify the eQTLs associated with HCM. Subsequently, the intersection of selected eQTLs and DEGs were plotted using the R package VennDiagram to obtain the key genes.

### 2.5 GO and KEGG enrichment analysis of DEGs between healthy control and HCM

The clusterProfiler and org.Hs.eg.db packages were utilized to conduct functional enrichment analysis on the DEGs (https://bioconductor.org/packages/release/data/annotation/html/org.Hs.eg.db.html), followed by visualization of the results using the ggplot2 and ggpubr packages. The adjust p-value “qvalueFilter” <0.05 was employed for screening GO and KEGG pathways, and only the top 10 GO and KEGG pathways were presented.

### 2.6 Expression levels of key genes and receiver operating characteristic curve analysis

The Wilcoxon test was employed to examine disparities in key genes between healthy individuals and those with HCM, followed by the generation of box plots for visualization. The “pROC” package was employed to generate ROC curves and calculate the area under the curve (AUC), assessing the diagnostic value of single key gene expression levels in HCM.

### 2.7 Machine learning and SHAP-based feature interpretation

To evaluate the ability of multiple key genes to discriminate HCM, we employed ten mainstream machine learning algorithms using the caret package (https://cran.r-project.org/package=caret) based on the merged cohort dataset. The algorithms included Partial Least Squares (PLS), Random Forest (RF), Decision Tree (DTS), Support Vector Machine (SVM), Logistic Regression, K-Nearest Neighbors (KNN), eXtreme Gradient Boosting (XGBoost), Gradient Boosting Machine (GBM), Artificial Neural Network (NeuralNet), Generalized Linear Model Boosting (glmBoost). These algorithms were used to construct diagnostic prediction models. Using the createDataPartition function, we randomly sampled 70% of the merged cohort as the training set, with the remaining 30% assigned as the test set. ROC curves were generated based on predicted probabilities, and the AUC was calculated using the pROC package to evaluate the classification performance of each machine learning model.

Among all machine learning models, the one with the highest AUC was selected as the optimal model and used for subsequent feature interpretation analysis. The permshap function was applied to compute SHapley Additive exPlanations (SHAP) values, quantifying the contribution of each feature gene to the model’s output. SHAP values quantify the contribution of each feature (gene) to the model’s prediction in a game-theoretic manner. A positive SHAP value indicates that the gene expression increases the model’s predicted probability of HCM, whereas a negative value indicates a protective or lowering effect on the predicted risk. The shapviz package was utilized to generate SHAP bar plots, bee swarm plots, and waterfall plots for representative samples, visualizing the relative importance and directional impact of key feature genes.

### 2.8 Analysis of key genes expression levels and immune cell infiltration in HCM patients

The CIBERSORT method ((https://cibersort.stanford.edu/)) was employed to calculate the relative proportions of 22 immune cell types in each HCM sample, followed by Spearman correlation analysis to investigate the associations between key genes and individual immune cell populations.

### 2.9 Construction of the HCM ceRNA network

We utilized key genes to predict the target miRNAs from databases including miRanda, miRDB, miRWalk, and TargetScan. Only those miRNAs predicted by all four databases were considered as potential target candidates of the key genes. After obtaining the final potential miRNAs, we utilized the spongeScan database for predicting miRNA-lncRNA targets. Subsequently, we constructed a ceRNA network comprising mRNA-miRNA-lncRNA interactions, and visualized the network using Cytoscape software (Version 3.10.1).

### 2.10 Potential drug target prediction of HCM key genes

The Drug-Gene Interaction database (DGIdb) website (https://www.dgidb.org/) was utilized for the prediction of potential drug targets associated with key genes, followed by visualization of the gene-drug targets using Cytoscape software.

### 2.11 mRNA expression levels in HCM and normal samples were measured using PCR assay

To validate the expression levels of the key genes identified in our study, we performed quantitative real-time PCR (qRT-PCR) on peripheral blood mononuclear cell (PBMC) samples collected from patients with HCM and healthy controls. Total RNA was extracted from the samples using the TRIzol reagent (Invitrogen, Carlsbad, CA, USA) according to the manufacturer’s instructions. The RNA concentration and purity were measured using a NanoDrop ND-1000 spectrophotometer (Thermo Fisher Scientific, Waltham, MA, USA). High-quality RNA samples with an A260/A280 ratio between 1.8 and 2.0 were selected for subsequent experiments.The RNA samples were reverse-transcribed into cDNA using the PrimeScript RT reagent Kit (Takara Bio, Kusatsu, Japan). The reverse transcription reaction was performed in a 20 µL reaction volume containing 1 µg of total RNA, 1µL of PrimeScript RT Enzyme MixI, 4µL of 5×PrimeScript Buffer, 1µL of RT Primer Mix, and 1µL of gDNA Eraser. The reaction was carried out at 37 °C for 15 minutes, followed by 85 °C for 5 seconds to inactivate the enzyme. Quantitative real-time PCR was performed using the SYBR Premix Ex Taq II kit (Takara Bio) on a CFX96 Real-Time PCR Detection System (Bio-Rad Laboratories, Hercules, CA, USA). The reaction mixture (20 µL) contained 10µL of 2×SYBR Premix Ex Taq II, 0.4 µL of forward primer (10 µM), 0.4 µL of reverse primer (10 µM), 2 µL of cDNA template, and 7.2 µL of ddH2O. The thermal cycling conditions were as follows: initial denaturation at 95 °C for 30 seconds, followed by 40 cycles of 95 °C for 5 seconds and 60 °C for 30 seconds. A melting curve analysis was performed to confirm the specificity of the PCR products. The relative expression levels of the target genes were calculated using the 2^-^▵▵Ct method, with GAPDH as the internal reference gene (**Table 1**).

**Table 1:** Primer sequences for qRT-PCR valizdation of key genes.

Gene	Forward primer sequence (5’->3’)	Reverse primer sequence (5’->3’)
RNF165	CACAGATGGTCGTCCATGAAA	CTTCGCTTCTTATACTTGTGGGG
SNCA	TGGTGAGCGAAACAGAAGCC	CCATAGCAACCTGCGTAATGAA
SRGN	AGGTTATCCTACGCGGAGAG	GTCTTTGGAAAAAGGTCAGTCCT
MARCO	CAGCGGGTAGACAACTTCACT	TTGCTCCATCTCGTCCCATAG
STEAP4	GGCTTTGGGAATACTTGGGTT	TGGACAAATCGGAACTCTCTCC
SIGLEC9	CCACATACCAAGAATTGCACCC	ACAGAGAGCCGGTGATGTTTAT
TKT	TCCACACCATGCGCTACAAG	CAAGTCGGAGCTGATCTTCCT
GAPDH	ACCACAGTCCATGCCATCAC	TCCACCACCCTGTTGCTGTA

### 2.12 Statistical analysis

All statistical analyses were performed using R software (version 4.3.1). Differential expression analysis between HCM and normal samples was conducted using the limma package. The Wilcoxon test was applied to assess expression differences of key genes. Mendelian randomization analysis was carried out using multiple complementary methods, including inverse variance weighting, MR-Egger, weighted median, weighted modal, and simple modal. A p value less than 0.05 was considered statistically significant. Correlations between gene expression and immune cell infiltration were evaluated using Spearman correlation analysis. Receiver operating characteristic curves were plotted to assess diagnostic performance, and area under the curve values were calculated. All visualizations were generated using ggplot2 and related packages.

## 3 Results

### 3.1 Differential expression analysis and GWAS analysis between healthy control and HCM

**Figure 1:**
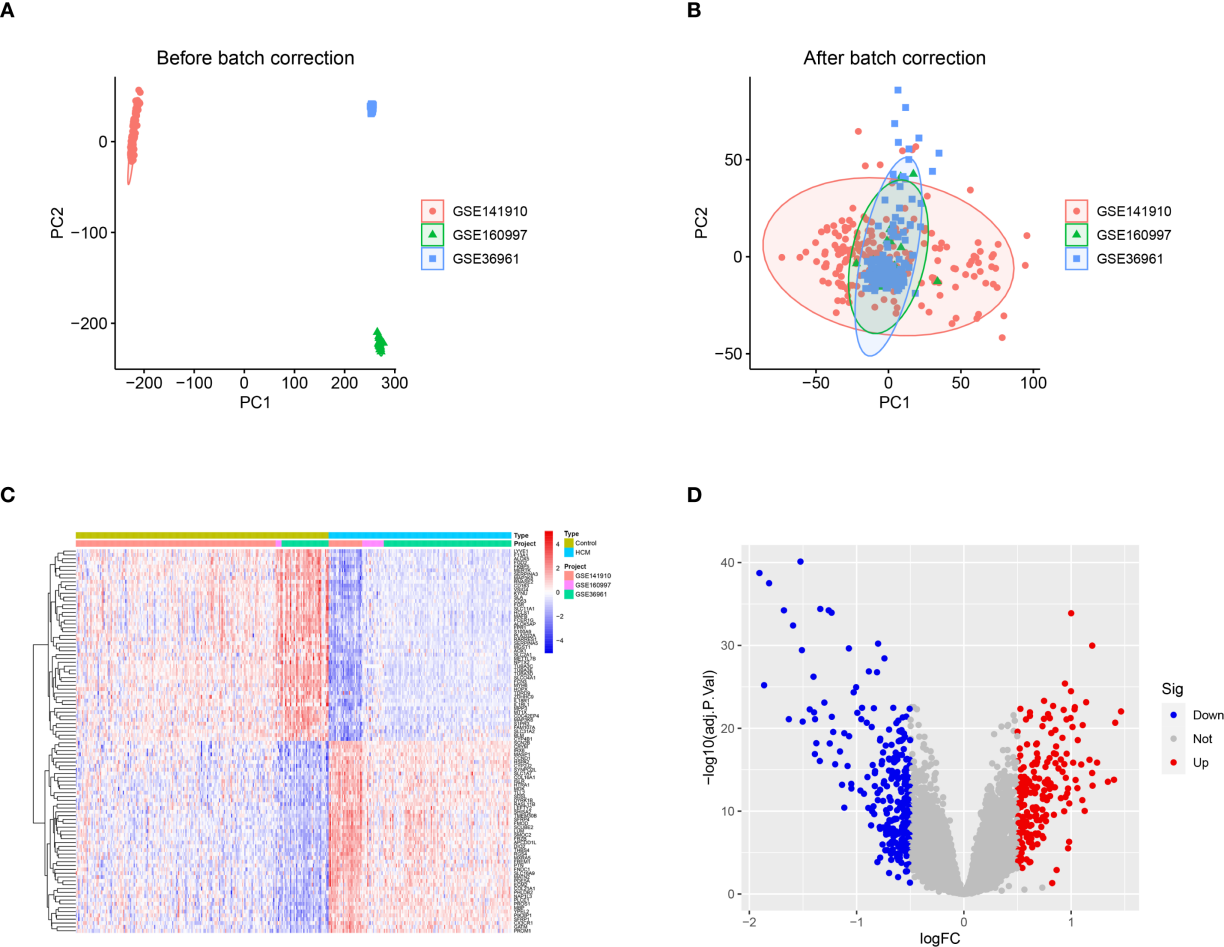
DEGs between healthy control and HCM. **(A)** The principal component analysis (PCA) was performed on the three data sets, PC1 and PC2, without prior standardization; **(B)** The principal component analysis (PCA) was performed on PC1 and PC2 after normalizing the data obtained from the three datasets; **(C)** Heat map of the top 50 DEGs between control and HCM; **(D)** Volcano plot of DEGs between control and HCM. DEGs, Differentially Expressed Genes; HCM, Hypertrophic Cardiomyopathy; PCA, Principal Component Analysis; PC1, Principal Component 1; PC2, Principal Component 2.

Following normalization and batch correction, PCA demonstrated effective mixing of samples from different datasets, supporting successful platform harmonization (**Figures 1A, B**). A total of 472 DEGs were identified (**Figures 1C, D**). We conducted GWAS analysis individually on 19,942 eQTLs and HCM cases (GWAS ID: ebi-a-GCST90018861). A total of 5,430 eQTLs (**Supplementary Table S2**) containing 25,472 SNPs (**Supplementary Table S3**) were identified based on the SNP screening criteria. The IVW method p<0.05 and heterogeneity analysis p>0.05 (**Supplementary Table S4**) were employed to select a total of 205 eQTLs (**Supplementary Table S5**) for subsequent analysis.

### 3.2 Acquisition and expression levels and chromosomal localization analysis of key genes in HCM

**Figure 2:**
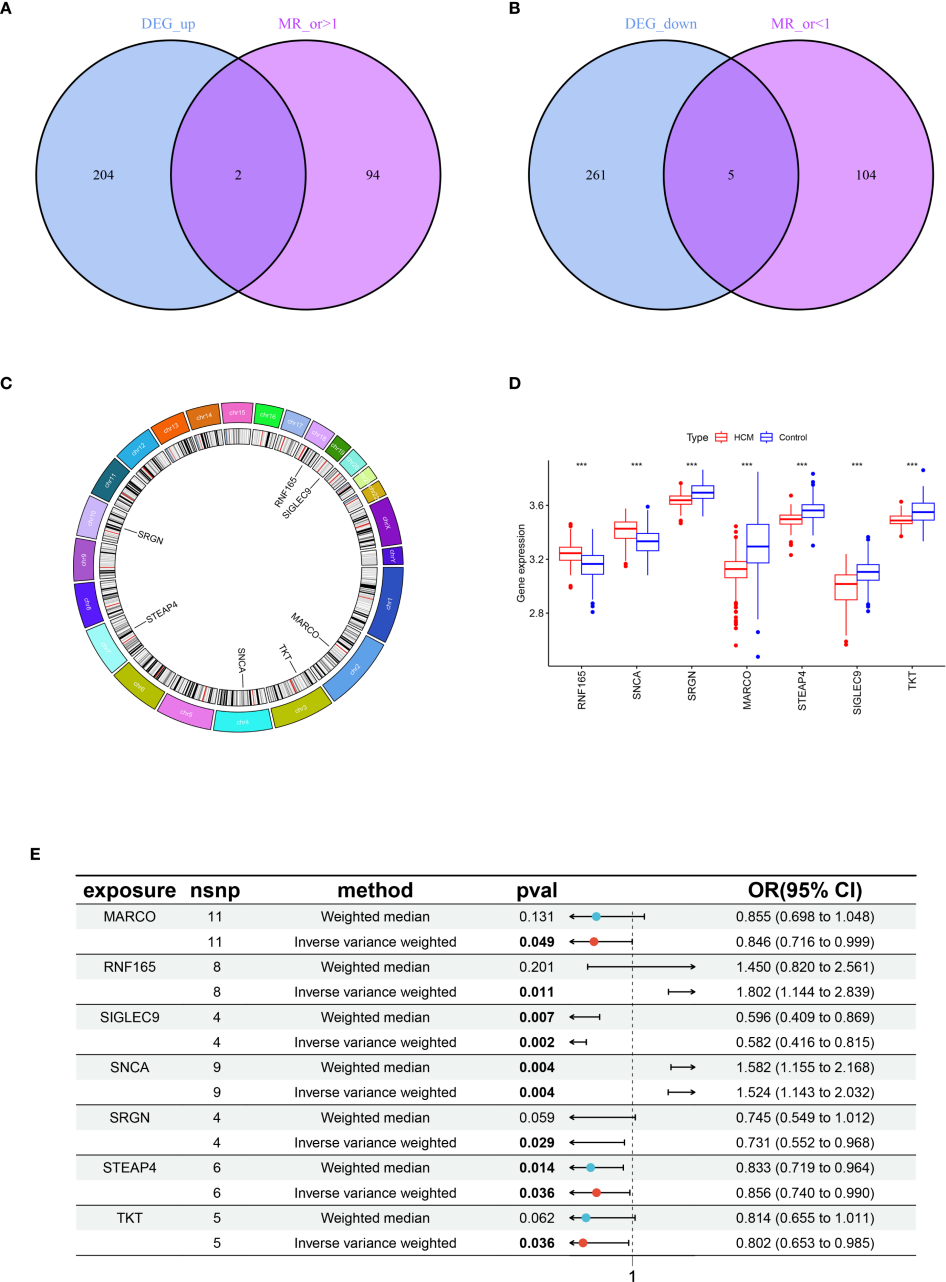
Acquisition and expression levels and chromosomal localization analysis of key genes in HCM. **(A)** Venn diagram of genes with intersection between highly expressed genes in HCM and MR analysis OR > 1; **(B)** Venn diagram of genes with intersection between lowly expressed genes in HCM and MR analysis OR < 1; **(C)** Chromosomal localization analysis of seven key genes; **(D)** Box plot of expression levels of seven key genes between healthy control and HCM, ***p < 0.001; **(E)** Forest plots illustrating the IVW and WM analysis methods for MR of seven key genes. HCM, Hypertrophic Cardiomyopathy; MR, Mendelian Randomization; OR, Odds Ratio; IVW, Inverse Variance Weighted; WM, Weighted Median.

Through integrative analysis of 472 DEGs and 205 eQTLs, two genes were found to be significantly upregulated and five genes significantly downregulated in HCM patients compared with control samples. Moreover, this elevated gene expression was associated with an increased incidence of HCM (MR_OR>1) (**Figure 2A**); Additionally, a set of 5 genes exhibited low expression levels in HCM (logFC<0), which was associated with an elevated risk of developing HCM (MR_OR<1) (**Figure 2B**). The highly expressed genes, Ring Finger Protein 165 (RNF165) and Synuclein Alpha (SNCA), are located on chromosomes 18 and 4 respectively; The lowly expressed genes, Serglycin (SRGN), Macrophage Receptor with Collagenous Structure (MARCO), Six Transmembrane Epithelial Antigen of the Prostate 4 (STEAP4), Sialic Acid Binding Ig Like Lectin 9 (SIGLEC9), and Transketolase (TKT) located on chromosomes10, 2, 8, 19, and 3 respectively (**Figures 2C, D**). The results of the MR analysis for the seven core genes were subsequently presented using a forest plot to illustrate the outcomes obtained from both IVW and WM methods (**Figure 2E**).

### 3.3 Results of GO and KEGG enrichment analysis of DEGs in HCM

**Figure 3:**
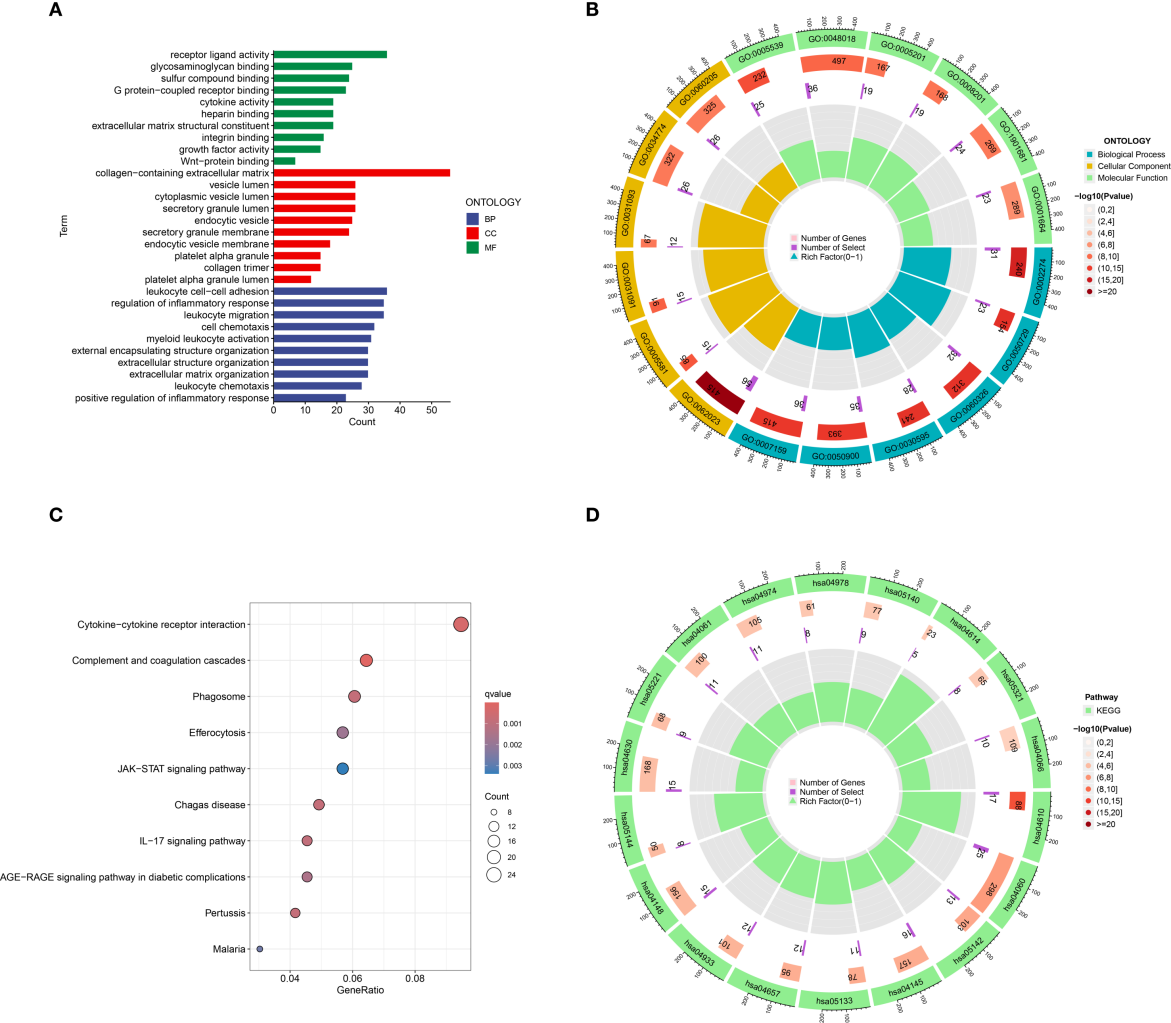
Go and KEGG functional enrichment analysis of DEGs in HCM. **(A)** The barplot of GO enrichment functions; **(B)** The circlize diagram of GO enrichment functions; **(C)** Bubble diagram of top 10 KEGG signaling pathways; **(D)** The circlize diagram of the signaling pathways. GO, gene ontology; KEGG, Kyoto Encyclopedia of Genes and Genomes; DEGs, Differentially expressed genes.

The results of the GO functional enrichment analysis indicated that receptor ligand activity, glycosaminoglycan binding, sulfur compound binding, G protein−coupled receptor binding, cytokine activity, heparin binding, extracellular matrix structural constituent, integrin binding, and Wnt−protein binding, growth factor activity were the top 10 ranks molecular functions (MFs); Collagen-containing extracellular matrix, vesicle lumen, cytoplasmic vesicle lumen, secretory granule lumen, endocytic vesicle, secretory granule membrane, endocytic vesicle membrane, platelet alpha granule, collagen trimer, and platelet alpha granule lumen were the top 10 rank cell components (CC); Leukocyte cell-cell adhesion, regulation of inflammatory response, leukocyte migration, cell chemotaxis, myeloid leukocyte activation, external encapsulating structure organization, extracellular structure organization, extracellular matrix organization, leukocyte chemotaxis, and positive regulation of inflammatory response were the top 10 rank biological processes (BP) (**Figures 3A, B**).

The results of the KEGG analysis indicated that Cytokine-cytokine receptor interaction, Complement and coagulation cascades, Phagosome, Efferocytosis, JAK-STAT signaling pathway, Chagas disease, IL-17 signaling pathway, AGE-RAGE signaling pathway in diabetic complications, Pertussis, and Malaria were the top 10 rank enrichment KEGG pathways (**Figures 3C, D**).

### 3.4 Machine learning based HCM diagnostic model with SHAP interpretation

**Figure 4:**
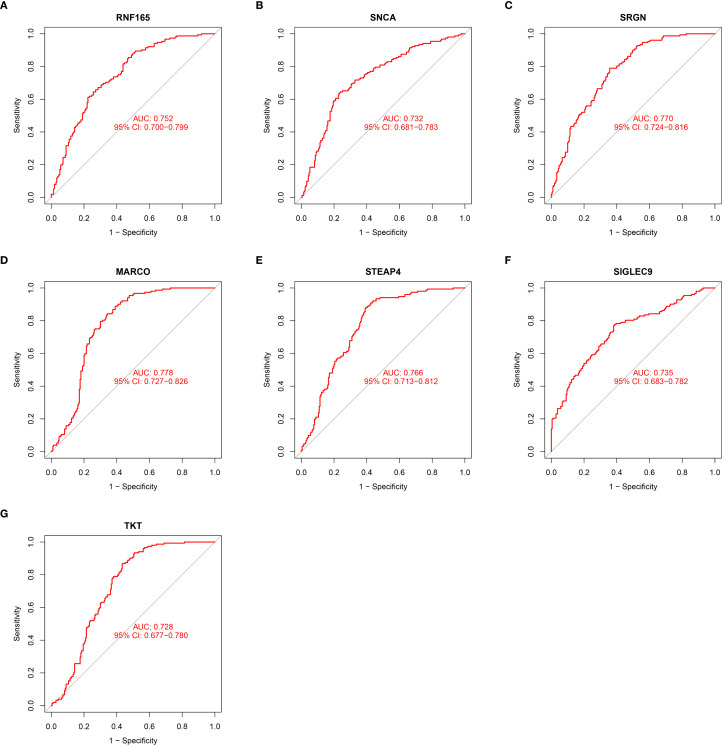
ROC curve illustrating the diagnostic potential of key gene expression levels in HCM patients. **(A)** RNF165; **(B)** SNCA; **(C)** SRGN; **(D)** MARCO; **(E)** STEAP4; **(F)** SIGLEC9; **(G)** TKT. RNF165, Ring Finger Protein 165; SNCA, Synuclein Alpha; SRGN, Serglycin; MARCO, Macrophage Receptor with Collagenous Structure; STEAP4, Six Transmembrane Epithelial Antigen of the Prostate 4; SIGLEC9, Sialic Acid Binding Ig Like Lectin 9; TKT, Transketolase.

At the single-gene expression level, the AUC values of the ROC curve for HCM patient diagnosis were RNF165 = 0.752, SNCA = 0.732, SRGN = 0.770, MARCO = 0.778, STEAP4 = 0.766, SIGLEC9 = 0.735, and TKT = 0.728 (**Figures 4A–G**).

**Figure 5:**
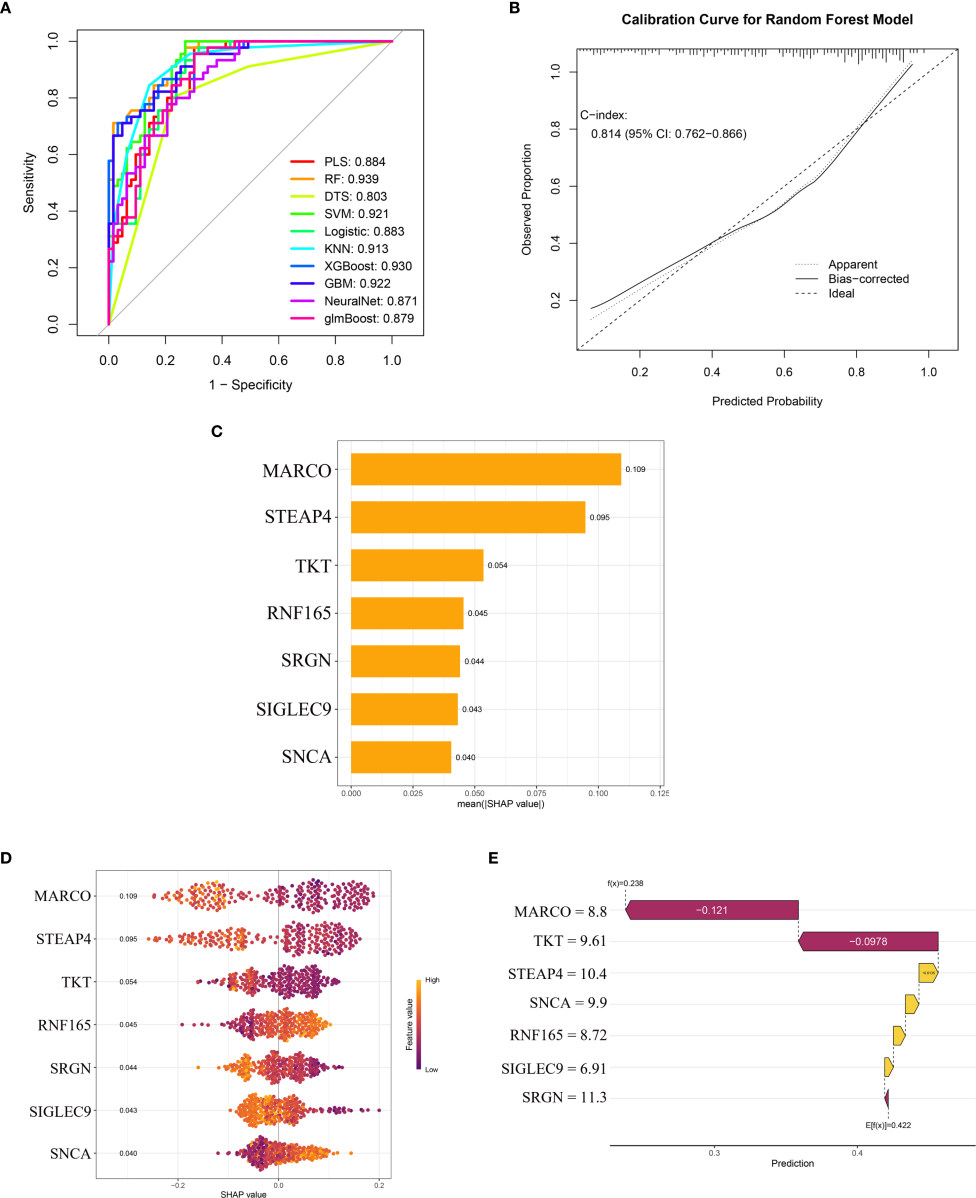
Machine learning model performance and SHAP-based interpretation of key genes in HCM diagnosis. **(A)** ROC curves of ten machine learning models; **(B)** The calibration curve of the random forest model; **(C)** SHAP summary bar plot showing the mean absolute contribution of key genes; **(D)** SHAP bee swarm plot displaying the distribution and directionality of gene effects; **(E)** SHAP waterfall plot for a representative sample. ROC, Receiver Operating Characteristic; SHAP, Shapley Additive Explanations; HCM, Hypertrophic Cardiomyopathy.

After incorporating seven key genes into ten machine learning algorithms, we found that the Random Forest (RF) model exhibited the highest AUC value of 0.939 (**Figure 5A**). Additional performance metrics for the RF model included an accuracy of 0.824, precision of 0.833, recall of 0.873, and an F1-score of 0.853, indicating favorable discrimination and predictive power. Furthermore, the calibration curve (**Figure 5B**) demonstrated good agreement between predicted and observed outcomes, with a C-index of 0.814, supporting the model’s robustness and potential clinical applicability. The SHAP summary bar plot revealed that MARCO contributed most significantly to the model’s predictive output, whereas SNCA had the smallest impact (**Figure 5C**).

The SHAP bee swarm plot (**Figure 5D**) displays the impact of the seven key genes on the Random Forest (RF) model’s predictions. MARCO exhibited the highest mean absolute SHAP value (0.109), indicating its dominant role in HCM discrimination, followed by STEAP4 (0.095) and TKT (0.054). Notably, RNF165 and SNCA showed positive SHAP values (mean: 0.045 and 0.040, respectively), suggesting that higher expression of these genes was associated with an increased predicted risk of HCM. In contrast, the remaining genes (MARCO, STEAP4, TKT, SRGN, SIGLEC9) demonstrated negative SHAP values, implying an inverse relationship with HCM likelihood (**Figure 5D**).

The SHAP waterfall plot further elucidates gene-specific effects (**Figure 5E**), deconstructing the model’s prediction for a representative sample. The baseline prediction value (E[f(x)] = 0.422) was adjusted based on each gene’s contribution, with values above 0.422 classified as HCM samples and values below 0.422 classified as normal controls. The expression levels of the seven genes in this sample were as follows:

MARCO (expression level = 8.8) exerted the strongest negative influence, significantly reducing the predicted HCM risk probability. TKT (9.61) and SRGN (11.3) further drove the prediction downward through negative SHAP values. In contrast, STEAP4 (10.4), SNCA (9.9), RNF165 (8.72), and SIGLEC9 (6.91) contributed positive effects, partially offsetting the impact of other genes. The final predicted value for this sample was f(x) = 0.238. According to the RF model algorithm, this sample was classified as normal, and the prediction was confirmed to be correct.

Although these biomarkers were identified in myocardial tissue, their diagnostic performance may not be directly translatable to clinical settings due to the impracticality of obtaining cardiac biopsies. Therefore, their current value lies in elucidating disease mechanisms.

### 3.5 Analysis results of key genes expression levels and immune cell infiltration in HCM patients

**Figure 6:**
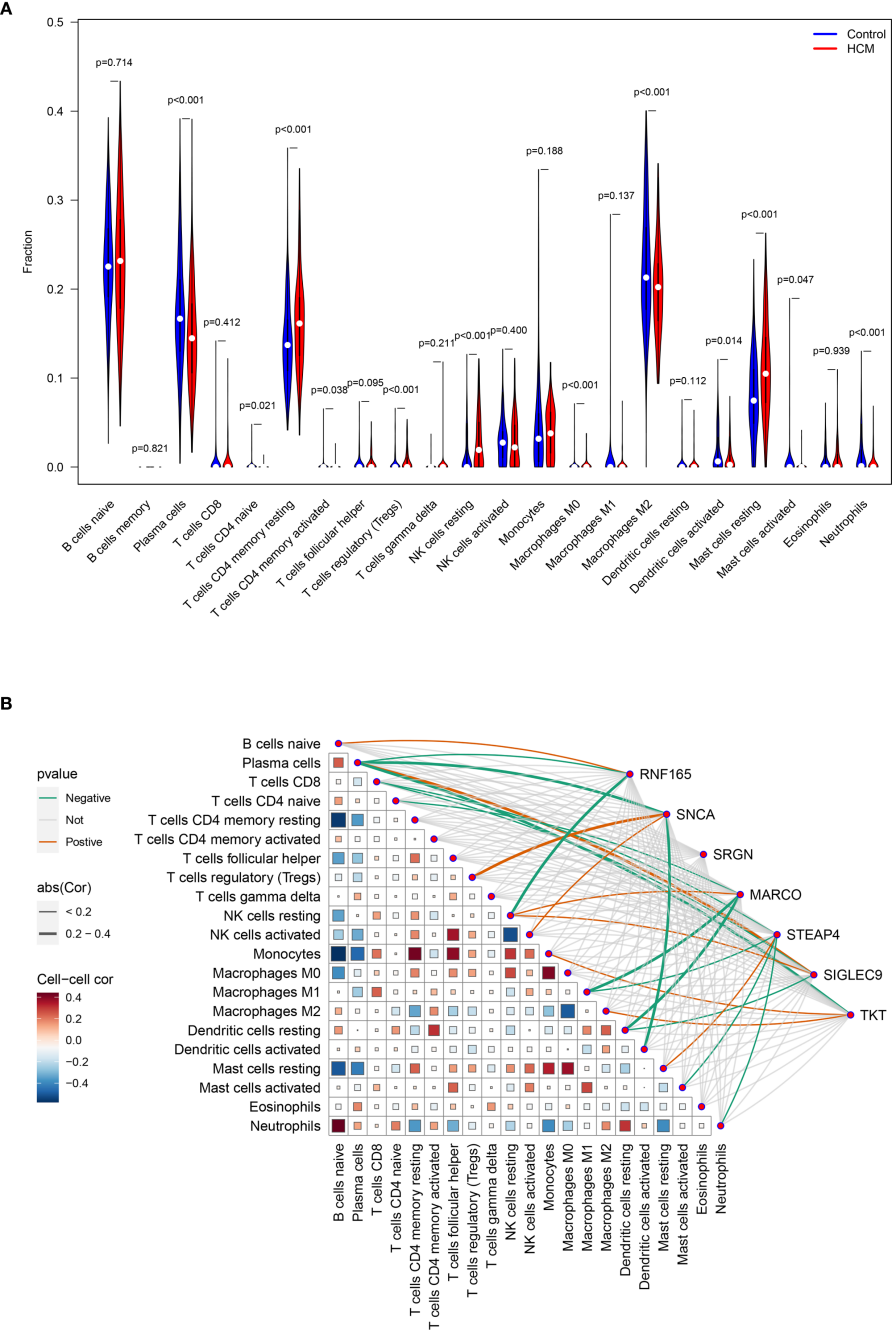
Analysis of key genes expression levels and immune cell infiltration in HCM patients. **(A)** Analysis of the differences in infiltration levels of 22 immune cells between healthy control and HCM patients; **(B)** Heat map for correlation analysis between seven key genes and 22 types of immune cells. HCM, Hypertrophic Cardiomyopathy.

Compared with the immune cell infiltration levels in the control group, four out of the 22 immune cell types exhibited significantly higher infiltration in HCM, while seven immune cell types showed lower infiltration in HCM. (**Figure 6A**).

The expression level of RNF165, SNCA, MARCO, STEAP4, SIGLEC9, and TKT was positively correlated with the infiltration degree of 1, 2, 1, 1, 2, 2 kinds of immune cells, respectively, and negatively correlated with the infiltration degree of 2, 2, 3, 4, 2, and 1 kinds of immune cells, respectively. There was no correlation between the expression level of SRGN and the degree of immune cell infiltration (**Figure 6B**).

### 3.6 Construction of the ceRNA network and potential drug target prediction of key genes

**Figure 7:**
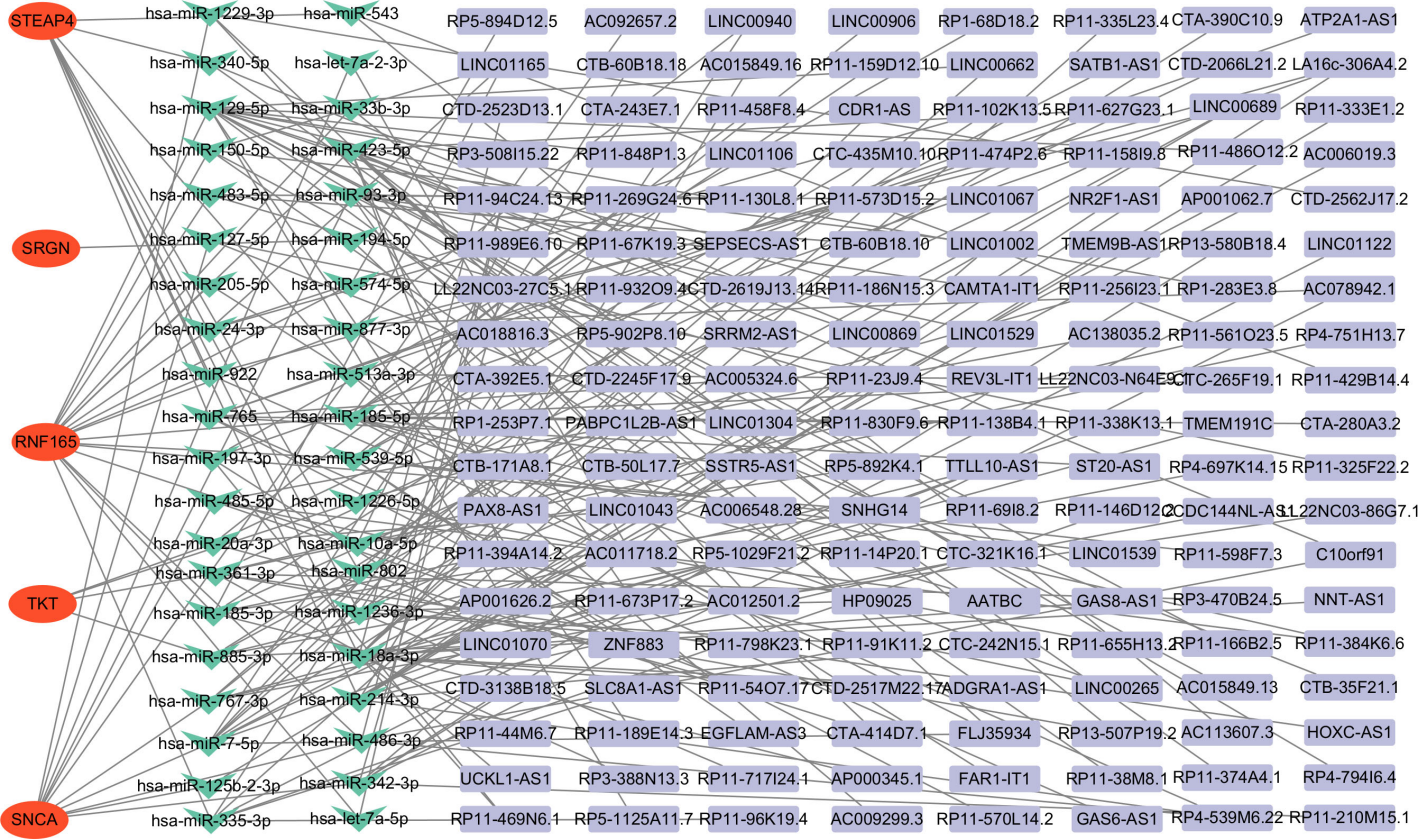
Construction of the ceRNA network in HCM patients. ceRNA, competing endogenous RNA, HCM, Hypertrophic Cardiomyopathy.

The ceRNA network was constructed by screening miRNAs and lncRNAs through online database systems, incorporating a total of 5 mRNAs, 40 miRNAs, and 152 lncRNAs. The network was visualized using Cytoscape. Among the seven key genes, five were included in the network: STEAP4, SRGN, RNF165, TKT, and SNCA (**Figure 7**).

**Figure 8:**
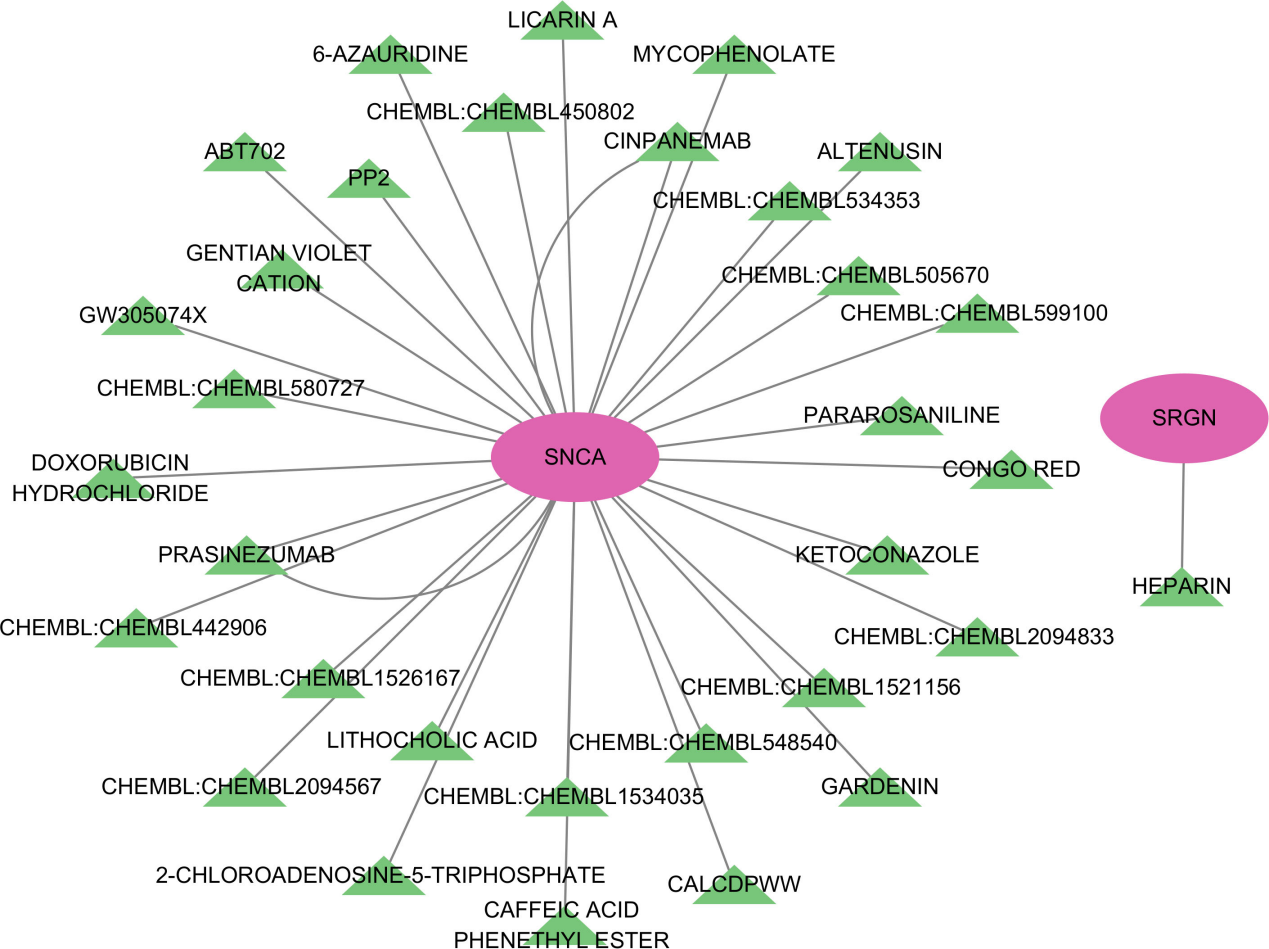
Network diagram of potential drug target prediction for key genes.

After conducting potential drug prediction of the 7 key genes in the DGIdb database, a total of 2 genes (SNCA and SRGN) were identified as potential targets for 34 drugs. Specifically, the SRGN gene was found to be targeted by HEPARIN drug, while SNCA was identified as a potential target for the remaining 33 drugs (**Figure 8**).

### 3.7 Validation of pCR levels in normal and HCM samples

**Figure 9:**
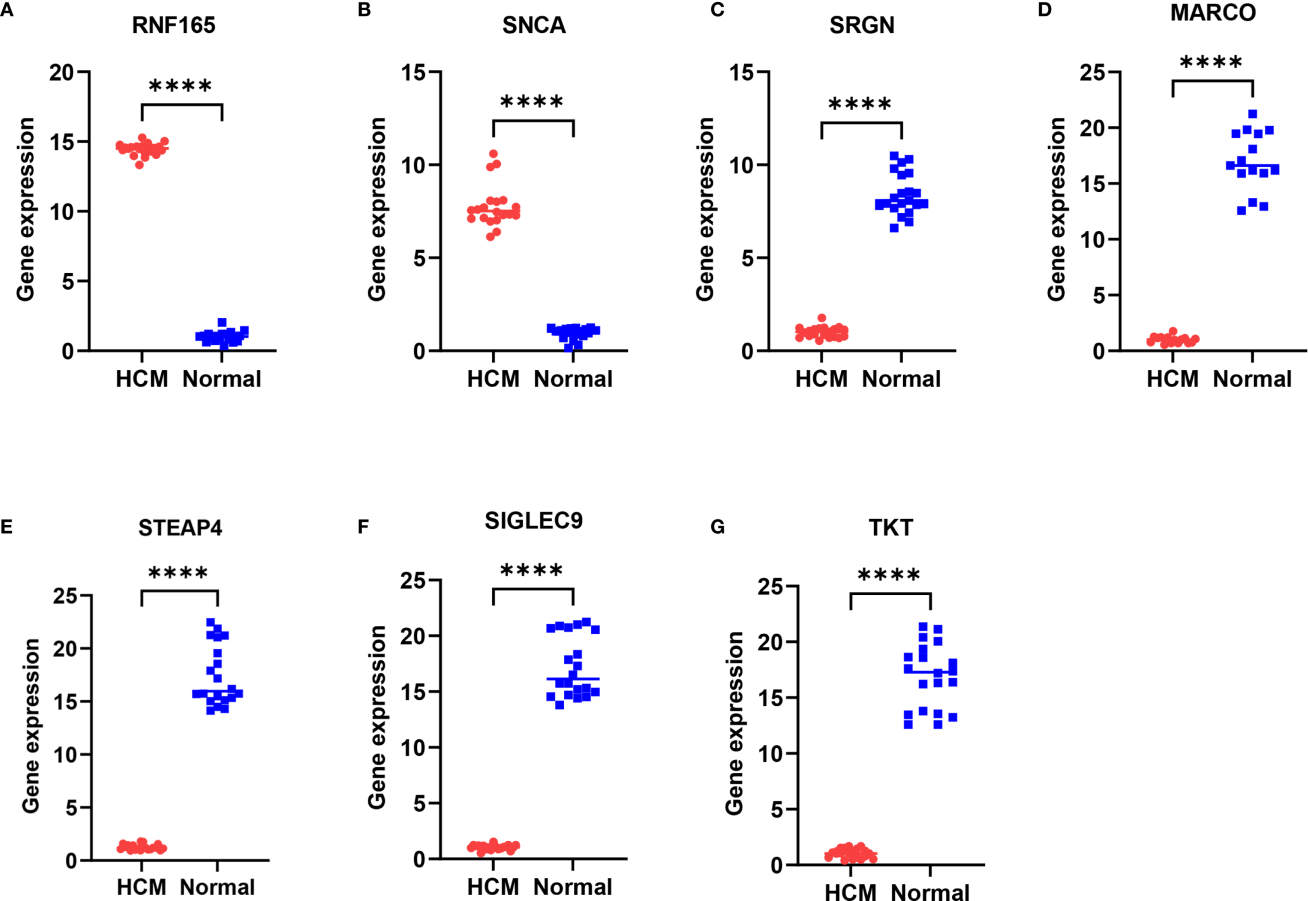
Differential expression of key genes in peripheral blood mononuclear cells between HCM patients and healthy controls. **(A)** RNF165; **(B)** SNCA; **(C)** SRGN; **(D)** MARCO; **(E)** STEAP4; **(F)** SIGLEC9; **(G)** TKT. ****p < 0.0001. RNF165, Ring Finger Protein 165; SNCA, Synuclein Alpha; SRGN, Serglycin; MARCO, Macrophage Receptor with Collagenous Structure; STEAP4, Six Transmembrane Epithelial Antigen of the Prostate 4; SIGLEC9, Sialic Acid Binding Ig Like Lectin 9; TKT, Transketolase.

A total of 35 subjects were included in this study, comprising 15 patients with hypertrophic cardiomyopathy (HCM) and 20 healthy controls. As shown in **Table 2**, there were no significant differences in age, sex distribution, or ejection fraction (EF) between the two groups (all p > 0.05). The results showed that compared with normal samples, RNF165 and SNCA were upregulated in HCM samples, while SRGN, MARCO, STEAP4, SIGLEC9, and TKT were downregulated (**Figures 9A–G**). These pCR results were consistent with the mRNA expression patterns observed in the GEO dataset.

**Table 2:** Baseline clinical and echocardiographic characteristics of HCM patients and controls.

Variable	Control (n = 20)	HCM (n = 15)	Z/χ²	P value
Age (years)	45.50 (36.5-55.75)	47.00 (42.00-58.00)	1.168	0.254
Ejection fraction (EF, %)	57.50 (55.25-60.75)	55.00 (54.00-60.00)	1.358	0.174
Maximum LV wall thickness (mm)	11.00 (10.25-12.00)	18.00 (16.00-19.00)	5.075	<0.001
Gender			1.944	0.163
Female	10	4		
Male	10	11		

Values are presented as median (interquartile range, IQR) for continuous variables and counts for categorical variables. Group comparisons were performed using the Mann–Whitney U test or χ² test, as appropriate.

## 4 Discussion

Hypertrophic cardiomyopathy is generally recognized as a monogenic heart disease that is an important cause of sudden arrhythmia death, heart failure, and atrial fibrillation (with embolic stroke). It is one of the common hereditary heart diseases in the world, but with the technological progress of sequencing technology and molecular biology, the sequencing results of HCM and further exploration of its pathogenesis cannot be used to explain the occurrence and development of HCM with a single gene mutation. Therefore, it is necessary to further study and analyze the genomic changes of HCM compared with healthy people in order to deeply understand its pathogenesis and disease heterogeneity.

Specifically, we summarize that seven immune-associated genes-RNF165, SNCA, MARCO, SIGLEC9, SRGN, STEAP4, and TKT-were identified using transcriptomic data, eQTL-based Mendelian randomization, and machine learning. These genes showed consistent expression trends and predictive potential in HCM. To comprehensively evaluate the roles of key genes in HCM diagnosis and immune regulation, we employed ten mainstream machine learning algorithms to construct diagnostic prediction models and interpreted each gene’s contribution through SHAP analysis. The Random Forest (RF) model demonstrated the highest AUC value among all algorithms, exhibiting superior diagnostic performance. These results confirmed that the significantly differentially expressed genes in HCM have good diagnostic value for HCM. Therefore, we further verified the results on human specimens and found that these genes in HCM human samples were consistent with the results of public data analysis. Tan Z et al. found that SNCA and TKT were significantly higher in the HMC model group than in the control group through public database transcriptome and experimental verification analysis results (24). Gu X et al. found that SRGN was enriched in HCM pathway (25) through long-term analysis of COVID in a comprehensive cohort of two years of proteomic exploration. RNF165, MARCO, SIGLEC9, and STEAP4 have not yet been reported in HCM. Rnf165/Ark2C enhances BMP-Smad signaling mediated motor axon elongation (26). MARCO and SIGLEC9 have been reported in many studies on immunity (27–29). The results of Dong Q et al. obtained the MARCO immunotherapy biomarker by analyzing the public database (27, 30–32). Another study suggests that targeting MARCO and IL37R on lung cancer immunosuppressive macrophages blocks regulatory T cells and supports cytotoxic lymphocyte function (28). These results indicate that MARCO is closely related to the immune system, and the immune system disorder plays an important regulatory role in HCM. SIGLEC9 positive tumor-associated macrophages predict prognosis and treatment vulnerability in colon cancer patients. Zheng Y systematically elucidates the role of natural killer cells SIGLEC7 and SIGLEC9 in viral infection and tumor progression (32). Specifically, we now state that while many functional insights about these genes originate from cancer or infectious disease models, accumulating evidence suggests that scavenger receptors (e.g., MARCO) and inhibitory checkpoint molecules (e.g., SIGLEC9) play conserved roles in tissue-specific immune responses, including the heart (33, 34). STEAP4 has been preliminarily studied in liver and prostate cancer (35, 36). Zhao J et al. confirmed that the expression of STEAP4 in resident cells of the central nervous system promotes Th17 cell-induced autoimmune encephalomyelitis (37).

While our study validates differential mRNA expression of immune-related genes such as MARCO and SIGLEC9, their functional roles in cardiac tissue remain poorly characterized. Based on prior studies, these genes are primarily expressed in infiltrating immune cells, particularly macrophages, rather than cardiomyocytes. In the context of the myocardium, MARCO-a scavenger receptor-is likely to be involved in monocyte/macrophage-mediated phagocytosis and immune suppression (38), while SIGLEC9 functions as an inhibitory receptor that may modulate T cell and NK cell activation (39). It is plausible that these genes contribute to the formation of an immunosuppressive microenvironment in HCM, potentially through canonical pathways such as IL-10 or TGF-β, both of which are known to promote fibroblast activation and myocardial fibrosis. However, our current study lacks single-cell or spatial transcriptomics data to directly distinguish between cell-type sources, and no proteomic or histological validation was performed to confirm pathway activation. Therefore, MARCO, SIGLEC9 and STEAP4 may affect HCM through the regulation of inflammation and immunity, and their exact molecular mechanisms in HCM still need to be further studied. Although myocardial transcriptomic profiling provides insights into local immune remodeling in HCM, we further validated the expression of key genes using peripheral blood samples, demonstrating their consistent expression patterns and highlighting their potential utility as non-invasive biomarkers in clinical practice. Future studies should aim to assess whether these immune-related genes or their corresponding protein products can be detected and quantified in plasma, serum, or circulating extracellular vesicles from HCM patients. Several recent studies have shown that immune-related cardiac biomarkers, such as IL-6, TGF-β, or galectin-3, are detectable in blood and correlate with myocardial remodeling and prognosis in cardiomyopathies (40, 41). Accordingly, peripheral validation and longitudinal follow-up are essential steps to determine the diagnostic or prognostic utility of these candidate markers.

Changes in genes are often accompanied by effects or alterations in biological processes that affect the course of disease, Therefore, we further carried out functional enrichment analysis on differential genes and the results showed that HCM compared with H1 in healthy group had significant differential expression of genes in cytokine activity, extracellular matrix structural constituent, integrin binding, and Wnt-protein binding, regulation of inflammatory response, leukocyte migration, myeloid leukocyte activation and the JAK-STAT signaling pathway, IL-17 signaling pathway, AGE- RAGE signaling pathway and other molecular signaling pathways as important components. The enrichment of JAK-STAT and IL-17 signaling is particularly relevant, as both pathways are known to mediate cardiac inflammation and fibrosis. JAK-STAT signaling—especially through IL-6 and IFN-γ—can promote macrophage activation and fibroblast proliferation, contributing to myocardial remodeling (42). IL-17, secreted mainly by Th17 cells, has been implicated in neutrophil-driven inflammation and extracellular matrix disruption in various cardiomyopathies (43). These findings suggest that dysregulation of these signaling cascades may be a common mechanism linking immune activation to structural changes in HCM. A review by Fang L et al. systematically explores the tumor necrosis factor (TNF)-α, interleukin (IL)-6 and serum amyloid P (SAP) were significantly increased in HCM patients compared to controls (18). Sewanan LR et al. ‘s findings reveal that the extracellular matrix of hypertrophic myocardium leads to impaired tic dynamics of healthy cardiomyocytes (44). RyR2 dysfunction mediated by Wang Y integrin β1D defect is one of the causes of catecholamine-sensitive ventricular tachycardia in arrhythmogenic right ventricular cardiomyopathy (45). It was also found that the regulation of inflammatory response also plays an important role in the occurrence and development of HCM. As for white blood cell migration, white blood cells are the main effector cells that produce inflammation in the body. As mentioned above, some core genes of differential expression of HCM are closely related to immunity, and they may play a regulatory role in the development of HCM by regulating immune response. Chatterjee S et al. confirmed that leukocyte telomere length is related to the severity of hypertrophic cardiomyopathy, and the shorter leukocyte telomerase is, the more severe HCM is (46). It is well known that the length of telomerase is related to the lifespan of cells, and its specific mechanism of action still needs further study. IL-17 and JAK-STAT molecular signaling pathways have not been studied in HCM, while JAK-STAT has been extensively studied in different types of cardiomyopathy. Prmt7 has a sex-specific cardioprotective effect by modulating the JAK/STAT signaling pathway (47). Sitapliptin alleviates cardiomyopathy in experimental diabetic rats by inhibiting the JAK/STAT signaling pathway (48). These results further confirm that the significantly differentially expressed genes in HCM play an important regulatory role in the pathogenesis of cardiomyopathy.

We have discussed above that the significantly differentially expressed genes in HCM may play important regulatory roles in immune and inflammatory responses and thus influence the progression of HCM. Therefore, we further analyzed the infiltration abundance of different types of immune cells and the interaction of significantly differentially expressed genes with immune cells in HCM and healthy controls. Compared to healthy samples Plasma cells, Macrophages M2, Dendritic cells activated, Neutrophils were lower infiltration in HCM samples, but T cells CD4 naive, T cells CD4 memory resting, T cells CD4 memory activated, T cells regulatory (Tregs), Macrophages M0, Dendritic cells resting were higher infiltration in HCM patient samples. Bioinformatics and immunoosmotic analysis reveal key pathways in the pathogenesis of hypertrophic cardiomyopathy and immune cells (49). Recent bioinformatics studies have increasingly highlighted the role of immune dysregulation in HCM. Hou et al. conducted a comprehensive analysis using necroptosis signatures and immune infiltration profiles, identifying elevated expression of pro-inflammatory pathways in HCM myocardium (16). Similarly, Zhang et al. demonstrated that key immune cell populations such as M2 macrophages and regulatory T cells are significantly altered in HCM tissues, supporting our own immune infiltration findings (49). Mononuclear RNA sequencing revealed changes in intercellular communication and dendritic cell activation in non-obstructive hypertrophic cardiomyopathy (50), results showing increased dendritic cell expression in HCM is inconsistent with this study. Becker RC et al. systematically described the potential role of the Von Willebrand factor and neutrophil extracellular trap in the natural history of hypertrophic and hypertensive cardiomyopathy (51). Yehia et al. ‘s findings revealed that the increased neutrophil-to-lymphocyte ratio was a negative prognostic indicator for HCM cats (52). The results of our analysis showed that various T cell subtypes generally showed a state of high infiltration in HCM. Shintani Y et al. studied the clinical effects of using endocardial biopsy to pathologically quantified myocardial fibrosis and infiltrating T lymphocytes in patients with hypertrophic cardiomyopathy (53). The greater the number of infiltrating CD3+ cells, the worse the clinical prognosis of HCM patients.

In addition to their immunomodulatory functions, several identified genes may also influence fibrotic remodeling, which is a hallmark of advanced HCM. For instance, immune cells such as macrophages and T-helper cells are known to secrete pro-fibrotic cytokines like TGF-β1, thereby promoting fibroblast activation and extracellular matrix deposition (54). Genes such as MARCO and STEAP4, which are involved in inflammatory signaling and redox regulation, may indirectly modulate this fibrotic cascade. These interactions suggest that immune dysregulation and fibrosis may be mechanistically linked in HCM pathogenesis. These results confirm that immune cells play an important role in the development of HCM. But a major limitation of our study is the reliance on bulk transcriptomic data, which cannot resolve gene expression at the single-cell level. Although we used CIBERSORT to estimate immune cell proportions, this method infers population averages and does not clarify the specific cellular sources of key genes such as MARCO or SIGLEC9. Recent studies using single-cell and spatial transcriptomics in HCM have revealed diverse immune cell subsets and complex intercellular communication. For example, Nie et al. reported enhanced dendritic cell activity and fibroblast–immune cell interactions in HCM myocardium (55), while Bos et al. demonstrated spatially restricted immune niches in cardiac tissue (56). These high-resolution approaches could help determine whether the immune-related genes we identified are expressed by infiltrating immune cells, resident cardiac macrophages, or other cell types. Future studies integrating single-cell and spatial data will be essential to validate our findings and clarify how immune cells contribute to myocardial remodeling in HCM. Besides, the lack of comparison with other cardiac pathologies such as dilated cardiomyopathy (DCM) or myocarditis, due to the absence of such samples in our dataset. This prevents us from determining whether the observed immune signatures are specific to HCM. However, prior studies have reported distinct immune profiles in different cardiomyopathies. For example, myocarditis is often marked by CD8+ T cell-mediated injury and dendritic cell activation, whereas DCM tends to show macrophage-driven inflammation and extracellular matrix remodeling (57). Future studies integrating transcriptomic data across multiple cardiomyopathy subtypes will be essential to determine the specificity and clinical utility of immune-related biomarkers in HCM. Notably, recent studies have suggested that sex-specific immune responses may influence the development and progression of hypertrophic cardiomyopathy. For example, female patients with HCM may exhibit heightened fibrosis, altered T-cell responses, and distinct macrophage activation patterns compared to males (58, 59). While our study lacked access to complete sex-stratified metadata from public datasets, we recognize this as a critical variable and recommend future studies to incorporate sex-based immunogenomic analyses to enhance precision in biomarker discovery and disease modeling.

The regulation of gene expression *in vivo* is influenced by various factors, including non-coding RNAs such as lncRNAs and miRNAs. To explore this, we constructed a ceRNA network centered on five core genes (RNF165, SNCA, SRGN, STEAP4, and TKT) identified in our study. The analysis revealed that these genes are potentially regulated by several non-coding RNAs, suggesting that ceRNA interactions may play a role in modulating immune and metabolic pathways in HCM. Previous studies have shown that non-coding RNAs participate in cardiovascular diseases by regulating oxidative stress, fibrosis, and immune activation. For example, SNCA has been linked to mitochondrial dysfunction and is a known miRNA target in neural and cardiac tissues (60). SRGN, involved in the storage and release of inflammatory mediators, may be regulated by lncRNAs under pro-inflammatory conditions (61). Although RNF165 is primarily studied in neurobiology (26), our findings suggest it may also be regulated by cardiac non-coding RNAs, potentially affecting inflammatory signaling in HCM. These findings highlight the possibility that ceRNA networks contribute to key processes in HCM such as immune infiltration, myocardial remodeling, and metabolic dysfunction. However, functional validation of these lncRNA–miRNA–mRNA interactions in cardiac tissue remains lacking. Future studies using cardiac-specific single-cell sequencing or RNA-interference experiments will be essential to clarify these regulatory mechanisms and their potential as therapeutic targets. The ultimate goal of all disease research always comes back to finding drugs to treat the disease, SRGN gene was found to be targeted by HEPARIN drug, SRGN gene was found to be targeted by heparin drug, while SNCA was identified as a potential target for the remaining 33 drugs, while SNCA was identified as a potential target for the remaining 33 drugs, These results indicate that these two genes may now serve as potential therapeutic gene targets for HCM. Similar to non-coding RNAs, there are drugs that target these core genes, but no studies have demonstrated the efficacy of these drugs against HCM. As mentioned above, these core genes participate in many important biological processes for the development of HCM, but their specific roles in HCM are not yet clear, so the specific therapeutic and biological effects of their targeted drugs and non-coding RNAs in HCM are also not yet known. Therefore, these results urgently require us to further verify these results and explore the specific biological regulation process of HCM. While our study identified 33 potential therapeutic agents targeting immune-related genes in HCM, translational application requires careful consideration. For example, heparin, although known for its anti-inflammatory properties, lacks specificity for myocardial tissue and carries bleeding risk. In contrast, agents like resveratrol and metformin have shown cardioprotective or anti-fibrotic effects in animal models of HCM and warrant further investigation (62, 63). We consulted DrugBank (https://go.drugbank.com/) to assess pharmacokinetic properties, cardiac safety, and therapeutic precedent. Several agents exhibited favorable oral bioavailability and cardiac safety profiles, but off-target immunosuppressive effects or systemic toxicity remain concerns. Future preclinical validation using cardiomyocyte and cardiac immune cell models is essential to prioritize candidates for clinical translation.nistic insights into post-translational regulation.

In summary, our study highlights several core genes that are not only differentially expressed in HCM but also significantly correlated with immune cell infiltration, suggesting a potential immunoregulatory mechanism underlying HCM pathogenesis. The construction of a ceRNA network revealed that these genes are regulated by multiple lncRNAs and miRNAs, although their roles in HCM remain largely unexplored and are currently better understood in cancer research. While our study validates differential expression of the seven key genes at the mRNA level using qRT-PCR, it does not assess protein expression or functional consequences. Given that transcript levels may not always correspond to protein abundance or activity, this represents a limitation of our current study. Future work should incorporate proteomic approaches (e.g., Western blotting, mass spectrometry) or immunohistochemistry to verify protein-level changes in myocardial tissues and to explore their spatial distribution and functional roles in HCM pathogenesis. Although our study establishes a strong association between immune-related genes and HCM, it remains unclear whether such immune activation is a cause or consequence of myocardial remodeling. While Mendelian randomization provides preliminary causal inference, definitive validation requires functional studies. Future work should employ gene knockdown or overexpression in animal models (e.g., cardiac-specific MARCO or SIGLEC9 transgenic mice), and CRISPR-based editing in human iPSC-derived cardiomyocytes. Single-cell and spatial transcriptomics combined with *in vivo* immune modulation may further elucidate whether immune activation precedes or follows structural cardiac changes in HCM. Furthermore, drug-gene interaction analysis suggested that SRGN may be targeted by heparin, and SNCA by over 30 candidate compounds, implying their potential as therapeutic targets. However, the effectiveness of these drugs in treating HCM is unknown due to the absence of experimental validation in cardiac-specific models. Considering that these genes are involved in immune-related processes such as cytokine signaling, leukocyte migration, and inflammatory regulation, future studies should focus on elucidating how gene expression modulates immune cell behavior and contributes to myocardial remodeling. Given the invasive nature of myocardial sampling, our identified markers should be considered mechanistic rather than directly diagnostic at this stage. Future work should aim to evaluate whether these genes-or their related protein or transcript levels-are detectable and discriminative in peripheral blood, plasma, or exosomal samples, which would facilitate their translation into clinically viable biomarkers. Taken together, our findings not only identify novel diagnostic and therapeutic targets for HCM but also emphasize the crucial role of immune dysregulation in its progression, providing a foundation for future mechanistic and translational research.

## 5 Conclusion

In this study, we identified seven key genes including RNF165, SNCA, MARCO, SIGLEC9, SRGN, STEAP4, and TKT that are differentially expressed in HCM and significantly associated with disease risk and diagnostic value. Mendelian randomization analysis further supported the causal relationship between gene expression and HCM susceptibility. These genes were also closely related to immune cell infiltration, suggesting they may influence HCM progression through immune regulation. Functional enrichment revealed their involvement in immune-related pathways such as cytokine signaling, leukocyte migration, JAK-STAT signaling, and IL-17 signaling. Immune infiltration analysis confirmed abnormal activation of multiple immune cell subtypes in HCM samples, particularly T cells and macrophages. Furthermore, ceRNA network analysis suggested that non-coding RNAs may regulate these genes, and drug-gene interaction analysis identified potential therapeutic compounds, especially for SRGN and SNCA. Although further experimental validation is needed, our results provide novel insights into the immune-related molecular mechanisms of HCM and suggest potential diagnostic markers and therapeutic targets for future translational research.

## Data Availability

Publicly available datasets were analyzed in this study. This data can be found here: We have listed the GSE number of the GEO dataset in methodology.
